# Settlement of Controversy between Professors Blackman and Bartholow

**Published:** 1868-10

**Authors:** 


### Settlement of Controversy between Professors Blackman and Bartholow.

The following correspondence explains itself, and we are most happy to announce the adjustment of differences between physicians of so high standing and great worth:

Prof. R. Bartholow, M. D., Dear Sir:

I understand that you are about to publish a pamphlet in reply to one issued by Prof. Blackman. The offensive personalities which have characterized this discussion, are unprofessional in themselves, and injurious to the Medical College of Ohio, in which you are both Professors.

I have, therefore, to request, that you withhold the publication of your pamphlet until a Committee of the Faculty, consisting of Profs. Graham, Comegys, and myself, shall have determined in what way this controversy shall terminate.

Very truly,

M. B. WRIGHT,
Dean Fac. Med. Col. Ohio.

The Committee of the Faculty, consisting of the undersigned, adjudge that the controversy between Profs. Blackman and Bartholow shall terminate, by a reply from Dr. Bartholow to Dr. Blackman's pamphlet—said reply to be free from personalities.

This reply is subjoined, and is considered by the Committee to be final. The Committee have the pleasure to state that both parties have agreed to this conclusion of a very unfortunate public controversy.

M. B. WRIGHT,
JAMES GRAHAM,
C. G. COMEGYS.

